# Supplementary material for: Developing internationally agreed core indicators for surveillance of preconception health: protocol for a consensus study
Source: PLoS One. 2026 Jun 16;21(6):e0342576. doi: 10.1371/journal.pone.0342576 (PMC13271456; doi:10.1371/journal.pone.0342576)
Supplement: S1 File — (PDF) [file pone.0342576.s001.pdf]

Supplementary File 1: Standardised guidance to conduct small-scale community-based surveys and interviews

The following guidance was provided for iCIPHE Alliance members to conduct a survey or interviews to obtain community views on what matters most to people before pregnancy and parenthood. Standardised survey questions and a comparable interview topic guide were used for consistency or could be slightly adapted to ensure locally relevance and acceptability.

Guidance:

International consensus study

As part of the International Core Indicators for Preconception Health and Equity (iCIPHE) Alliance, we are conducting an international consensus study to develop a core set of indicators that can be used for surveillance of preconception health in low-, middle- and high-income countries. The study consists of the following steps:

- Step 1: Identifying an initial long-list of candidate surveillance indicators, and defining principles for scoring the importance of each indicator, through a literature review, public involvement, and workshop with iCIPHE Alliance members;
- Step 2: Scoring each candidate indicator in terms of its importance for surveillance through a two-round Delphi survey among study participants;
- Step 3: Reaching agreement on the final core indicator set through a series of consensus meetings with a select group of study participants.

Public involvement

As part of step 1, we are seeking iCIPHE Alliance members or international colleagues who can obtain local input from members of the public to ensure that the Delphi survey captures indicators and scoring principles that are relevant and important to people of reproductive age internationally.

Target group

In each World Health Organization (WHO) region[1], we aim to obtain input from 10-20 members of the public. The target group includes people of all genders, aged 18-49, from various backgrounds (i.e. diverse socio-demographic characteristics), irrespective of previous pregnancy experiences and future pregnancy and parenthood aspirations.

Input through online anonymous survey or discussion group

Based on suggestions from members of the public in the UK, we propose that input from the public is obtained through 1) **online anonymous surveys** or 2) **discussion groups**, depending on local circumstances, budget and existing groups that can be readily accessed.

[1] World Health Organization (WHO) regions: African Region; Region of the Americas; South-East Asian Region; European Region; Eastern Mediterranean Region; Western Pacific Region.

1. **Online anonymous survey**
Together with members of the public in the UK, we have developed an online survey (see below). The survey can be sent to your local public involvement group(s) or public connections to obtain input from 5-10 people. We ask that you do not share the survey on social media or through large mailing lists.

   The survey is developed in English, and may need translation into your local language. If the survey can be conducted in English, we can share a link and collect responses directly. Alternatively, you can re-create the survey using the questions below and send us the responses. Similarly, if you translate the survey into your local language, we can create a link for you to share, or you may create your own survey.

   The survey questions and answer options can be adjusted to ensure they are locally relevant and acceptable.

2. **Discussion group**
Based on the survey questions developed with members of the public in the UK, we have created a topic guide (see below) to facilitate discussion with 5-10 members of your local public involvement group(s) or public connections. These group discussion sessions can be in person or online, and are expected to take up to one hour. In the UK, public involvement sessions are not recorded or analysed, but notes are taken by the discussion facilitator(s) to capture the key findings (guidance included below). A member of our research team may be able to help facilitate a session if the discussions are in English.

   If relevant, please translate the topic guide into your local language, and adjust any questions or prompts to ensure they are locally relevant and acceptable.

Reimbursement

In the UK, members of the public who contribute to informing our research are often reimbursed for their time (for example through online shopping vouchers). Please follow your local guidelines for reimbursement. We would ask you to identify local funding for this, however, where needed we may be able to financially support reimbursement for members of the public who complete the online survey or participate in a discussion group.

Ethical approval requirements

In the UK, public involvement (or patient and public involvement, PPI) is an approach used to gain insights from the public to inform what research is relevant to them, how it should be conducted, and how findings are best disseminated. Members of the public thereby help *inform* the research rather than *participate* in the research as a study participant. Public involvement is therefore not classified as research and does not require ethical approval in the UK. Ethical approval requirements may differ across countries and organisations, so please consider if approvals are needed through your local organisation.

Ongoing public involvement

We have applied for further funding to set up an international public involvement group to support the international consensus study throughout the project, from informing the survey through to dissemination of the findings. We have therefore included a question at the end of the survey and discussion group topic guide to ask if members of the public consent to being contacted about further opportunities to contribute to our international project.

Anonymous online survey:

The questions below ask about things that you may find important to have in place or change before a potential pregnancy in the future. We are also interested in *how* you decide what is most important to you.

This survey is anonymous, which means that we don't collect any personal information from which you can be identified, such as your name and address. At the end of the survey we ask for your email address, but this is optional and will only be used for the purpose indicated in the specific questions.

Your answers will help us find out how we can best describe people's health before pregnancy and parenthood and look at whether this improves over time across different countries.

Thank you!

1. Imagine you were currently hoping to become pregnant*, what would be the most important factors for you to have in place before the pregnancy? (list as many factors as you like)

2. Imagine you were currently hoping to become pregnant*, are there any health-related factors you would want to change to increase the chances of a healthy pregnancy?

* *If it is your partner who would become pregnant, please still answer this question thinking about factors that are relevant to you, not necessarily your partner.*

--next page--

3. Thinking of what matters most to you to have in place before pregnancy, please rank these groups of factors from most to least important (1 = most important; 7 = least important)

- Mental health
- Physical health
- Money
- Living conditions
- Relationships and family
- Work/education
- Climate change

4. How did you decide which factors you put at the top of your list?

--next page--

A few questions about you:

5. Age:

6. Country of residence:

7. Ethnicity:

- White
- Mixed
- Asian
- Black
- Other:
- Prefer not to say

8. Sex:

- o Female
- o Male
- o Prefer not to say

9. Gender:

- o Woman
- o Man
- o Non-binary
- o Transgender
- o Other:
- o Prefer not to say

10. How is your health in general:

- o Very good
- o Good
- o Fair
- o Bad
- o Very bad

--next page--

11. Email address (only used to send you an e-voucher to thank you for completing the survey):

12. Your responses will help inform an international survey. Do you consent to receiving information from us about further opportunities to contribute to our international project on health before pregnancy and parenthood?

- o Yes
- o No

13. If yes, please enter your email address here:

Topic guide for facilitating a discussion group:

Suggested outline:

1. Introductions
2. Background to the discussion
3. Questions
4. Brief anonymous survey to collect key demographic information
5. End of session

1. Introductions

Introduce session facilitator(s) and ask all participants to briefly introduce themselves.

2. Background to the discussion

Provide brief information about the study:

We are developing an international research study which aims to find out how we can best describe people's health before pregnancy and parenthood and look at whether this improves over time across different countries.

You can help us develop this study through discussions about things that are important to have in place or change before a potential pregnancy in the future, based on your opinion. We are also interested in how you decide what is most important to you. We will use this to develop the research survey.

This session will take about 1 hour, we are interested in your personal opinions, there are no right or wrong answers, and we ask that you respect the opinions and thoughts of others. After this session we will send you a shopping voucher as a thank you for your contribution.

Any questions before we start?

3. Questions

Questions	Notes to take by facilitator(s)
Imagine you were currently hoping to become pregnant, what would be the most important factors for you to have in place before the pregnancy?<br>- Explain that if it is their partner who would become pregnant, the question relates to factors that are relevant to them, not necessarily their partner.<br>- Prompts (only if needed) – examples: social factors, behavioural factors, medical factors.	- *List all factors identified during the discussion.*<br>- *Where relevant, describe if certain factors were mentioned more often/considered more important than other factors.*
If you were currently hoping to become pregnant, are there any health-related factors you would want to change to increase the chances of a healthy pregnancy?<br>- Explain that if it is their partner who would become pregnant, the question relates to factors that are relevant to them, not necessarily their partner.	- *List all health-related factors identified during the discussion.*<br>- *Where relevant, describe if certain health-related factors were mentioned more often/considered more important than other factors.*

- Prompts (only if needed) – examples: achieving a healthy weight or stopping smoking.	
When thinking about the list of factors that are the most important for you to have in place before getting pregnant, where does your mental or physical health come on your list of priorities?	- *Summarise answers, e.g. health comes at the top, middle or bottom of the list.*
Where do other factors, such as money, living conditions, relationships and family, work/education, climate change (+ other factors mentioned in the discussion) come?	- *Summarise answers, e.g. which factors come at the top, middle or bottom of the list.*
How did you decide which factors you put at the top of your list?<br>Why are the factors at the top of your list more important than others?	- *Summarise reasons given for why factors were put at the top/bottom of the list.*
Any other comments or thoughts about important factors to have in place before getting pregnant, how you decide what these factors are or anything else you would like to add?	

4. Brief anonymous survey to collect key demographic information

Please complete a short survey with a few questions about you (survey link can be posted in the chat function if using an online platform). This survey is anonymous, which means that we don't collect any personal identifying information about you such as your name and address. At the end of the survey we ask for your email address, which is optional and will only be used for the purpose indicated in the specific questions.

(survey to be created based on questions 5-13 in the anonymous online survey)

5. End of session

- Thank participants for their contributions
- Ask if participants have any final questions
- Inform participants how they will receive a voucher/reimbursement
