# Supplementary material for: Developing internationally agreed core indicators for surveillance of preconception health: protocol for a consensus study
Source: PLoS One. 2026 Jun 16;21(6):e0342576. doi: 10.1371/journal.pone.0342576 (PMC13271456; doi:10.1371/journal.pone.0342576)
Supplement: S1 Table — (PDF) [file pone.0342576.s002.pdf]

Supplemental Table 1. Potential recruitment channels for the Delphi survey

Stakeholder groups for each WHO region	Recruitment channels
1. Community members and third sector/voluntary sector/charity representatives	
African Region	Ethiopia: Arba Minch University, Hawassa University, Wachamo University
Region of the Americas	Canada: Action Canada for Sexual Health & Rights<br>US: Black Mamas Matter Alliance<br>US: Black Women's Health Imperative<br>US: Health Connect One<br>US: MotherToBaby.org
South-East Asian Region	Bangladesh: BRAC (NGO focused on alleviating poverty and empowering the poor)<br>Bangladesh: Reproductive Health Services Training and Education Program (RHSTEP)<br>Bangladesh: Grameen Bank<br>India: SEWA/SEWA Bharat<br>India: Ekjut<br>Nepal: HERD international<br>Nepal: Helen Keller International (HKI) Nepal
European Region	UK: Patient and Public Involvement groups<br>Netherlands: www.zwangerwijzer.nl<br>Netherlands: www.Slimmerzwanger.nl<br>Netherlands: Patient Alliances<br>Netherlands: Consortia Pregnancy and Birth
Eastern Mediterranean Region	Qatar: Primary Health Care Corporation (PHCC)<br><br>Qatar: Qatar University<br>Pakistan: Community Health Sciences, Aga Khan University<br>Pakistan: Institute of Health and Development, Aga Khan University
Western Pacific Region	Australia: Fair agenda<br>Australia: Centre of Research Excellence in Health in Preconception and Pregnancy (CRE HiPP) Consumer Advisory Group<br>Australia: SPHERE Consumer Advisory Group<br>Japan: TUNAGARI (social networking service for students at Hokkaido University)<br>Singapore: SingHealth Duke-NUS Maternal and Child Health Research Institute
2. Health and social care professionals	
Global	International Confederation of Midwives
African Region	Ethiopia: Ethiopian Midwives Association
Region of the Americas	Canada: Society of Obstetricians & Gynecologists of Canada<br>Canada: Canadian Association of Midwives<br>Canada: Canadian Partnership for Maternal and Child Health<br>US: American College of Obstetricians & Gynecologists (ACOG)<br>US: Association of Maternal & Child Health Programs (AMCHP)<br>US: Dr. Laurie Zephryin<br>US: Dr. Diana Ramos<br>US: San Ysidro Health<br>US: Moore Institute for Nutrition and Wellness

Stakeholder groups for each WHO region	Recruitment channels
	US: Society of Family Planning<br>US: American Academy of Family Physicians<br>US: American Academy of Pediatrics<br>Brazil: medical societies such as FEBRASGO and nurse societies such as ABENFO<br>Latin American Center for Perinatology, Women and Reproductive Health (CLP/WR)
South-East Asian Region	Bangladesh: Bangladesh Medical Association (BMA)<br>Bangladesh: Bangladesh Society of O&G<br>South Asia Federation of O&G (SAFOG)
European Region	UK: Primary Care Women's Health Forum<br>UK: WISE GP<br>UK: UK Preconception Partnership<br>Netherlands: Royal Dutch Midwifery organisation<br>Netherlands: Dutch society of Obstetrics and Gynaecology<br>Netherlands: Dutch Society of General practitioners<br>Netherlands: Public Healthcare (GGD-GHOR)<br>Belgium: Flemish Organisation of Midwives<br>Belgium: alumni from postgraduate preconception and fertility care
Eastern Mediterranean Region	Qatar: Primary Health Care Corporation (PHCC)
Western Pacific Region	Australia: Royal Australian College of General Practitioners<br>Australia: Australian College of Midwives<br>Australia: National Preconception Health Network<br>Australia: Australian Primary Health Care Nurses Association<br>New Zealand: Royal Australian and New Zealand<br>New Zealand: College of Obstetricians and Gynaecologist (including He Hono Wāhine)<br>Japan: Japan Medical Association<br>Japan: Japan Academy of Midwifery<br><br>Japan: Preconception Health Network Japan<br>Singapore: Academy of Medicine Singapore (AMS)<br>Singapore: O&G Society of Singapore (OGSS)<br>Singapore: College of Family Physicians Singapore
3. Policy and programme professionals	
Global	WHO/UNICEF international and regional offices
African Region	Ethiopia: Ministry of Health
Region of the Americas	Canada: Public Health Agency of Canada’s<br>Canada: Canadian Perinatal Surveillance Systems Expert Advisory Committee (CPSS-EAC)<br>Canada: Statistics Canada’s Sexual and Reproductive Health Data Initiative Expert Advisory Committee (SRH-EAC)<br>US: CDC and HRSA<br>US: Association of Maternal & Child Health Programs<br>US: March of Dimes<br>US: American Heart Association<br>US: BRIDGE (Better Research, Information and Data Generation for Empowerment)

Stakeholder groups for each WHO region	Recruitment channels
	US: Maternal Health Learning and Innovation Center at the University of North Carolina at Chapel Hill
	Brazil: Brazilian National and Regional Primary Health Care Offices
South-East Asian Region	Bangladesh: Directorate General of Health Services (DGHS)
	Bangladesh: Ministry of Health and Family Welfare (MoHFW)
	UNICEF South Asia Region – country officer
European Region	UK: UK Preconception Partnership
	UK: Government Office for Health Improvement and Disparities
	Belgium: Study Center for Perinatal Epidemiology/Cepip
	Netherlands: PPS Solid Start
Eastern Mediterranean Region	Qatar: Ministry of Public Health (MOPH) – WHO office
Western Pacific Region	Australia: Public Health Association of Australia
	Australia: SPHERE Coalition
	Australia: National Preconception Health Network
	Japan: Children and Families Agency, Setagaya-Ward, Sapporo City
	Japan: Japanese Society of Public Health
	Singapore: Health Promotion Board (HPB)
	Singapore: Ministry of Social and Family Development (MSF)
	Singapore: People's Action Party Women's Wing
4. Researchers	
Global	Preconception and Interconception PubMed Update global email list
African Region	Ethiopia: Arba Minch, Hawassa University, Wachamo University
Region of the Americas	Canada: North American Primary Care Research Group, Contraception & Abortion Research Team
	National research groups focussed on reproductive life planning and chronic conditions and preconception health
	Brazil: The International Center for Equity in Health (ICEH) at the Federal University of Pelotas
South-East Asian Region	Bangladesh: Department of Public Health - North South University
	Bangladesh: Dhaka University, icddr,b (International Centre for Diarrhoeal Disease Bangladesh)
	Bangladesh: Public Health Foundation Bangladesh
	Population Council Consulting, Society for Applied Studies
European Region	UK: UK Preconception Partnership
	Netherlands: PPS Solid Start
	Netherlands: Nationwide institute for Health (Trimbos Institute)
	Netherlands: Nationwide institute for Public Safety and Milieu (RIVM)
Eastern Mediterranean Region	Qatar: Department of Public Health, College of Health Sciences, Qatar University
Western Pacific Region	Australia: National Preconception Health Network
	Australia: Health in Preconception, Pregnancy and Postpartum (HiPPP) Early- and Mid-career Researcher Collective (EMR-C)
	Australia: SPHERE Centre of Research Excellence in Women's Sexual and Reproductive Health in Primary Care
	New Zealand: Te Tātai Hauora o Hine – National Centre for Women's Health Research Aotearoa (Aotearoa New Zealand)

Stakeholder groups for each WHO region	Recruitment channels
	Japan: Preconception Health Network Japan (National Center for Child Health and Development, Hokkaido University, St. Lukes international university)<br>Singapore: SingHealth Duke-NUS Maternal and Child Health Research Institute
